# Increased representation of the non-dominant hand in pianists demonstrated by measurement of 3D morphology of the central sulcus

**DOI:** 10.1093/psyrad/kkab004

**Published:** 2021-05-28

**Authors:** Adam Harvey, Lewis Hou, Kirsteen Davidson-Kelly, Rebecca S Schaefer, Sujin Hong, Jean-François Mangin, Katie Overy, Neil Roberts

**Affiliations:** Reid School of Music, Alison House, 12 Nicolson Square, University of Edinburgh, EH8 9DF, UK; School of Clinical Sciences, The Queen's Medical Research Institute (QMRI), University of Edinburgh, EH16 4TJ, UK; School of Clinical Sciences, The Queen's Medical Research Institute (QMRI), University of Edinburgh, EH16 4TJ, UK; Scottish Chamber Orchestra, 4 Royal Terrace, Edinburgh, EH7 5AB, UK; Health, Medical and Neuropsychology Unit, Institute for Psychology, Leiden University, Leiden, The Netherlands; Academy of Creative and Performing Arts, Leiden University, Leiden, The Netherlands; Neuropolitics Research Lab and Edinburgh Imaging, School of Social and Political Science, University of Edinburgh, EH8 9LN, UK; Université Paris-Saclay, CEA, Centre National de la Recherche Scientifique (CNRS), Neurospin, Baobab, Gif-sur-Yvette, France; Reid School of Music, Alison House, 12 Nicolson Square, University of Edinburgh, EH8 9DF, UK; School of Clinical Sciences, The Queen's Medical Research Institute (QMRI), University of Edinburgh, EH16 4TJ, UK

**Keywords:** asymmetry, brain plasticity, central sulcus (CS), magnetic resonance imaging (MRI), motor cortex, musicians, pianists, surface area (SA)

## Abstract

**Background:**

Post-mortem and magnetic resonance imaging (MRI) studies of the central sulcus, as an indicator of motor cortex, have shown that in the general population there is greater representation of the dominant compared to the non-dominant hand. Studies of musicians, who are highly skilled in performing complex finger movements, have suggested this dominance is affected by musical training, but methods and findings have been mixed.

**Objective:**

In the present study, an automated image analysis pipeline using a 3D mesh approach was applied to measure central sulcus (CS) asymmetry on MR images obtained for a cohort of right-handed pianists and matched controls.

**Methods:**

The depth, length, and surface area (SA) of the CS and thickness of the cortical mantle adjacent to the CS were measured in each cerebral hemisphere by applying the BrainVISA Morphologist 2012 software pipeline to 3D T1-weighted MR images of the brain obtained for 15 right-handed pianists and 14 controls, matched with respect to age, sex, and handedness. Asymmetry indices (AIs) were calculated for each parameter and multivariate analysis of covariance (MANCOVA), and post hoc tests were performed to compare differences between the pianist and control groups.

**Results:**

A one-way MANCOVA across the four AIs, controlling for age and sex, revealed a significant main effect of group (*P* = 0.04), and post hoc analysis revealed that while SA was significantly greater in the left than the right cerebral hemisphere in controls (*P* < 0.001), there was no significant difference between left and right SA in the pianists (*P* = 0.634). Independent samples t-tests revealed that the SA of right CS was significantly larger in pianists compared to controls (*P* = 0.015), with no between-group differences in left CS.

**Conclusions:**

Application of an image analysis pipeline to 3D MR images has provided robust evidence of significantly increased representation of the non-dominant hand in the brain of pianists compared to age-, sex-, and handedness-matched controls. This finding supports prior research showing structural differences in the central sulcus in musicians and is interpreted to reflect the long-term motor training and high skill level of right-handed pianists in using their left hand.

## Introduction

Studies of the central sulcus (CS), which lies adjacent to the motor cortex, over several decades and using a range of methods have frequently (White *et al*., 1994; Amunts *et al*., 1996, 1997; Cykowski *et al*., 2008), although not always (White *et al*., 1997), shown a greater representation of the dominant compared to the non-dominant hand in the human brain. Findings of increased asymmetry with age (Cykowski *et al*., 2008) and decreased asymmetry with bilateral hand use (Li *et al*., 2010; Sun *et al*., 2012) suggest that that there may be a number of factors that affect CS asymmetry. Pianists, who spend years in training, performing complex motor skills with both hands, are an ideal cohort for further study. Here we report the application of an automated magnetic resonance (MR) image analysis protocol to measure the depth, length, and surface area (SA) of the CS, the average thickness of the cortical mantle on either side of the CS, and asymmetry of these measures, in a cohort of pianists and age-, sex-, and handedness-matched non-musician controls.

Analysis of postmortem adult brains, in which the extent of the anterior and posterior walls of the CS was traced on digitised images of cut sections, showed evidence of leftward asymmetry of the CS in the general population (assumed to be predominantly right handed) (White *et al*. 1994) but was not confirmed in a follow-up study (White *et al*., 1997). The first *in vivo* study was performed by Amunts *et al*. (1996), who analysed MR images obtained for 31 right-handed and 14 left-handed men for whom handedness was assessed using a hand dominance test (HDT) (Steinmetz *et al*., 1991; Steingrüber, 1971). The depth of the CS was measured by tracing the posterior wall of the precentral gyrus bordering the CS on horizontal section images (intra-sulcal length of precentral gyrus, ILPG) and was found to be greater in the left compared to the right cerebral hemisphere in the right-handed, and vice versa in the left-handed, male participants. Subsequently, Amunts *et al*. (2000) performed a similar MRI study for a larger cohort of 52 women and 51 men, with handedness assessed using the HDT and a 12-item questionnaire (Annett, 1970). The depth of the CS (determined via ILPG) was found to be significantly greater in the left compared to the right cerebral hemisphere in male participants assessed as being consistently right-handed. In contrast, in male participants assessed as being consistently left-handed no significant corresponding rightward asymmetry was observed. Furthermore, no inter-hemispheric asymmetry was found in either consistently right-handed or consistently left-handed females.

More recent studies have used MR image analysis techniques to reconstruct a 3D mesh representing the complex shape of the CS. Using this approach, Cykowski *et al*. (2008) reported finding significant leftward asymmetry of CS depth in both female (*n* = 28) and male (*n* = 27) participants, and which was located more superiorly in males. Sun *et al*. (2012) used a 3D mesh approach to compare the shape of the CS in 34 subjects born left-handed who had been forced to write with their right hand (i.e. forced right-handers), with 23 age- and sex-matched natural right-handers and 18 similarly matched natural left-handers. Left-handers forced to write with their non-preferred right hand displayed a pattern of CS SA asymmetry that is typical of natural right-handers. However, additional analysis revealed that the forced right-handedness did not cause a shift in the location of the so-called ‘hand knob’ (also referred to as the Omega Sign, and a major landmark of the representation of the hand in the motor cortex of the brain), which was situated more dorsally in the left hemisphere in natural right-handers compared to both natural left-handers and natural left-handers who had been forced to write with their right hand. Thus it would appear that cortical morphology in adults holds an accumulated record of both innate features and motor experience.

Up until now, to the best of our knowledge, there have been three studies of CS asymmetry in musicians. Amunts *et al*. (1997) measured the depth of the CS (measured via the ILPG as described above) in the left and right cerebral hemisphere of MR images obtained for 21 male keyboard players and 30 male controls. Controls exhibited a significant leftward CS asymmetry, but in the keyboard players there was no significant asymmetry, due to greater ILPG in the right, non-dominant cerebral hemisphere. Bangert and Schlaug (2006) obtained MR images for 32 musicians (keyboard and string players) and 32 age- and sex-matched controls and performed a study in which neuroanatomically experienced observers made a visual inspection on a gross anatomical scale of the appearance of the CS hand knob in each cerebral hemisphere. Ratings of a pronounced hand knob were significantly more frequent in expert musicians than controls, especially in the left cerebral hemisphere in keyboard players and in the right cerebral hemisphere in string players, an effect which was interpreted as being related to the specific motor demands of the different instruments.

Li *et al*. (2010) manually outlined the boundary of the CS on a mesh computed from 3D MR images and reported no significant difference in the length or SA of either the anterior or posterior walls of the CS in a comparison of pianists (all at student levels) (*n* = 20) and controls (*n* = 21). However, the pianists showed greater local variability of SA in the middle section (i.e. the somatotopic hand area) of the right CS and the lower portion of the left CS compared to controls. Additional analysis revealed a significant negative correlation between the variability of SA of the middle section of right CS and age of commencement of musical training, suggesting plasticity of the 3D morphology of the CS in pianists in response to long-term motor skill learning.

In the present study, similar to Cykowski *et al*. (2008) and Sun *et al*. (2012), an automated 3D mesh approach, available in the BrainVISA sulcal extraction and identification pipeline (http://brainvisa.info) was used to compare the morphology of the CS in pianists and controls. In particular, the depth, length, and SA of the CS and the average thickness of the cortical mantle on either side of the CS were measured for pianists and a group of age-, sex-, and handedness-matched controls. Based on previous research findings, the CS was predicted to show leftward asymmetry in controls but not in pianists, and this effect was predicted to be more sensitively detected by the two-dimensional SA measure than by the one-dimensional measures of depth, length, and cortical thickness.

## Materials and Methods

### Participants

The study cohort comprised 15 pianists (mean age 32.1 years, SD 11.6 years; 7 men) and 14 age- and sex-matched controls (mean age 31.2 years, SD 10.0 years; 7 men) (see Table 1). All participants were right-handed, scoring greater than 40 on the 10-item Edinburgh Handedness Inventory (Oldfield, 1971) (see Table 1). The pianists had all begun piano training before the age of 9 years (mean starting age 6 years; range 2-8 years). Seven were currently working as professional pianists, and the other eight were either in the final year of a piano training course at tertiary level or had recently completed training to this level. On average the pianists had played the piano for 26.3 years (range 13-50 years), whereas the control participants had an average of 1.9 years of training on any single musical instrument (range 0-7 years). None of the participants were known to have any neurological disorders. Approval was obtained from the Local Research Ethics Committee and all participants gave fully informed written consent.

**Table 1:** Participants’ mean age, score on the 10-item Edinburgh Handedness Inventory (EHI) and number of years of music training and results of analysis of significant differences using independent samples t-tests.

	Pianists (*n* = 15)		Controls (*n* = 14)		
	Mean	SD	Mean	SD	*P*
**Age**	32.1	11.6	31.2	10.0	0.821
**EHI score**	74.5	18.4	83.4	13.8	0.149
**Years of music training**	26.3	11.8	1.9	2.0	< 0.001

### MRI acquisition and analysis

**Figure 1:**
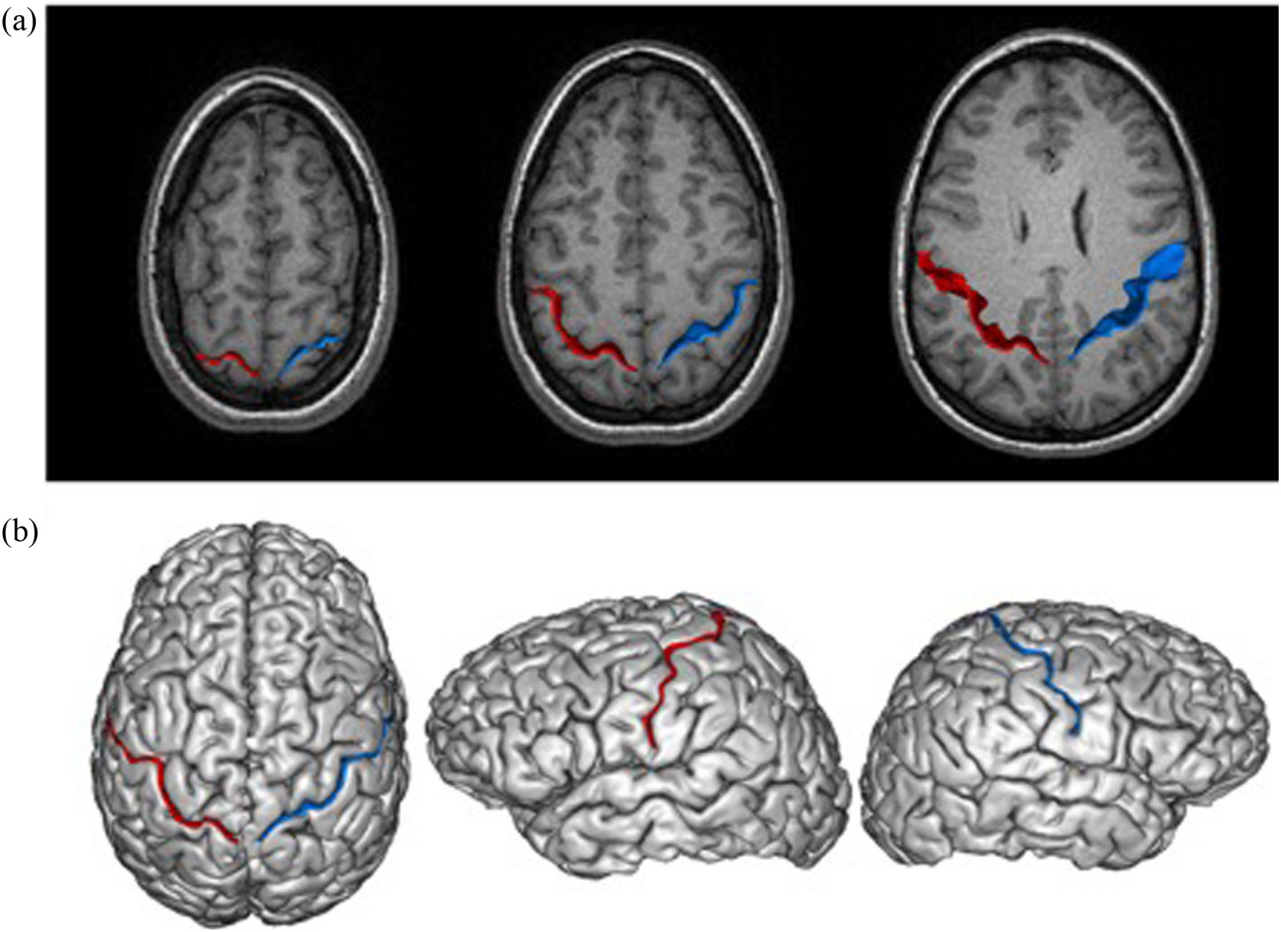
The image analysis pipeline is illustrated for an individual pianist. The 3D MR image was processed using the Morphologist 2012 image analysis pipeline in BrainVISA software. This enables segmentation and labelling of the CS in the left and right cerebral hemisphere, shown in red and blue, respectively, in each of the panels. The measurements of depth, length, and SA refer to the sulcal ribbon shown cutting through a systematic series of transverse MRI images in panel (a). In (b), the intersection of the ribbon with a 3D rendering of the brain is shown for superior and lateral (left and right hemispheres) views, respectively.

MRI investigations were performed at the Edinburgh Imaging facility in the Queen's Medical Research Institute, University of Edinburgh. For each participant a 3D T1-weighted image was obtained using a 3 T Verio MRI system (Siemens Healthineers, Erlangen, Germany) with the following acquisition parameters: repetition time (TR) 2300 ms, echo time (TE) 2.98 ms, inversion time (TI) 900 ms, flip angle 9°, field of view (FOV) 240 mm × 256 mm, matrix 240 × 256, 160 slices and slice thickness 1 mm. The 3D images were transferred to the analysis workstation and blinded with regard to group status, and also with respect to left–right orientation by flipping randomly. The resulting images were imported into BrainVISA software (http://brainvisa.info) and analysed using the Morphologist 2012 pipeline. The process (see Mangin *et al*., 2004; Mangin *et al*., 2010) comprised skull stripping, correction for inhomogeneity of image signal intensity, normalisation to Talairach space using anatomical landmarks, correction for image signal intensity inhomogeneity, and image segmentation. Next, the cerebrum was separated from the cerebellum and divided into left and right cerebral hemispheres, and sulcal folds were automatically detected and labelled using the SPAM algorithm (Statistical Probabilistic Anatomy Map) (Perrot *et al*., 2011) (see Fig. 1). For one of the participants (later revealed as one of the controls and excluded from analysis, reducing the group size from 15 to 14) the image analysis pipeline could not be applied successfully. The resulting sulcal fold ‘ribbons' were independently viewed in Talairach space and checked for quality by two observers with training in neuroanatomy using the integrated Anatomist tools, and any discrepancies in labelling were manually amended according to consensus reached following discussion. Three parameters were computed from the 3D mesh of the CS in the left and right cerebral hemispheres using the BrainVISA Morphometry Statistics tool, namely, depth (average), length, and SA, plus the average thickness of the cortical mantle adjacent to the CS. More details regarding the measures can be found in Kochunov *et al*. (2012).

### Statistical analysis

All statistical analyses were performed using SPSS version 26 (IBM Corp. Released 2019. Armonk, NY, USA). Asymmetry indices (AIs) were calculated for the depth, length, SA, and cortical thickness values measured within each cerebral hemisphere for each individual using the formula AI = (Right - Left)/(0.5 × (Right + Left)), which corrects for inter-individual variation in overall size (Toga and Thompson, 2003) and where positive values indicate rightward asymmetry and negative values indicate leftward asymmetry. All within-hemisphere measurements and resulting AIs were shown to be continuous, linear, and normally distributed by analysis of kurtosis, and equality of variances was confirmed by Levene’s test.

To investigate the effect of group differences in the four AIs, a one-way multivariate analysis of covariance (MANCOVA) was performed, with age and sex as covariates. Next a 2 × 2 mixed design MANCOVA was performed for the measured depth, length, SA, and cortical thickness values (between-subject factor: musicians, controls; and within-subject, repeated measures factor: left hemisphere, right hemisphere) with age and sex as covariates. For each MANCOVA, post hoc analyses were performed using ANCOVAs and t-tests to identify the source of any effects. All univariate analyses were performed at an alpha level of 0.05 (Bonferroni corrected) and with 2-tailed t-tests.

## Results

**Figure 2:**
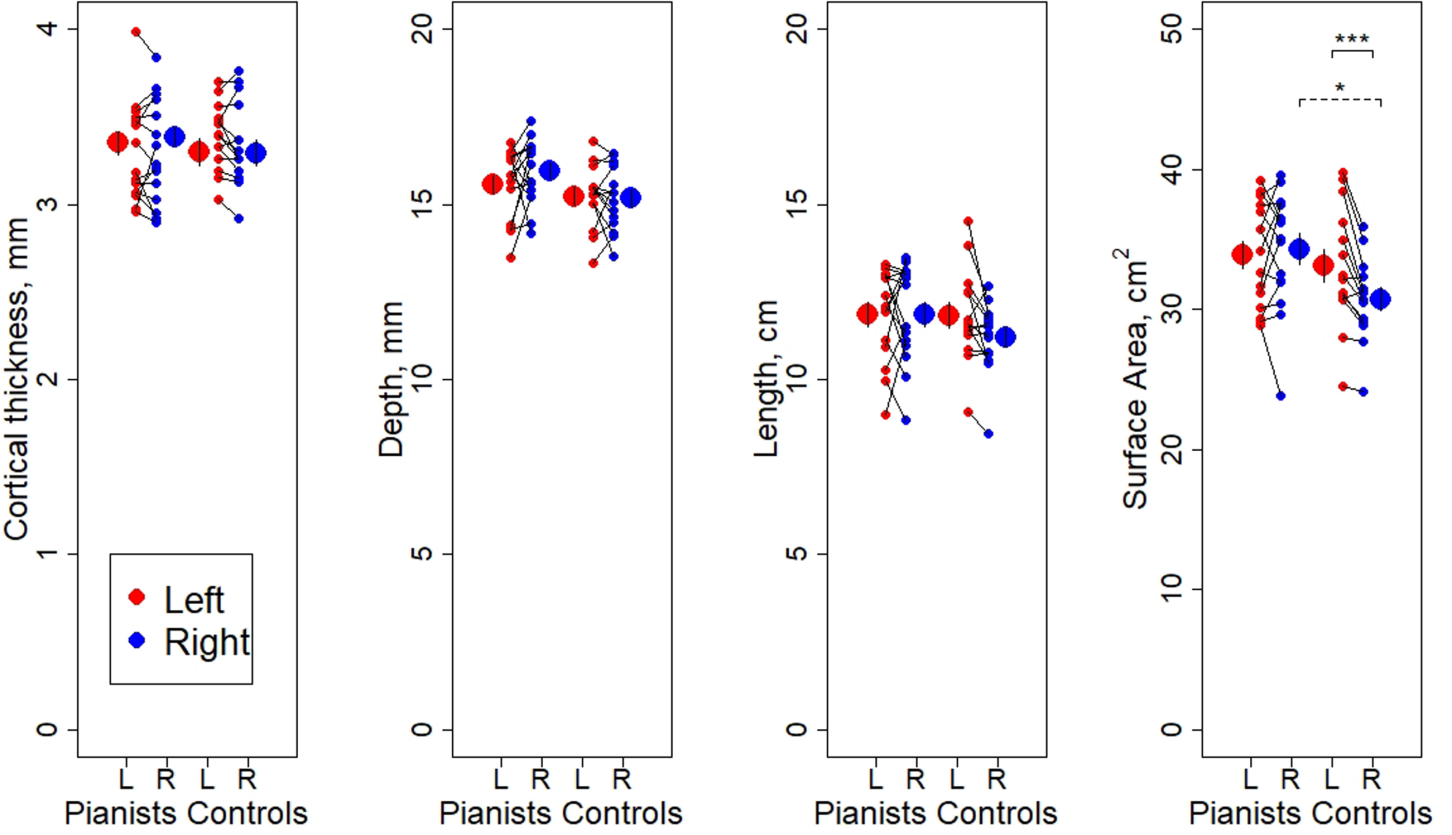
Individual data points and mean values of cortical thickness, depth, length, and SA of the CS in pianists (left side of each panel) and controls (right side of each panel). Statistically significant findings for independent samples t-test between groups and paired t-test within groups are denoted by * for *P*< 0.05 and *** for *P* < 0.001. Error bars represent ± two standard errors.

**Figure 3:**
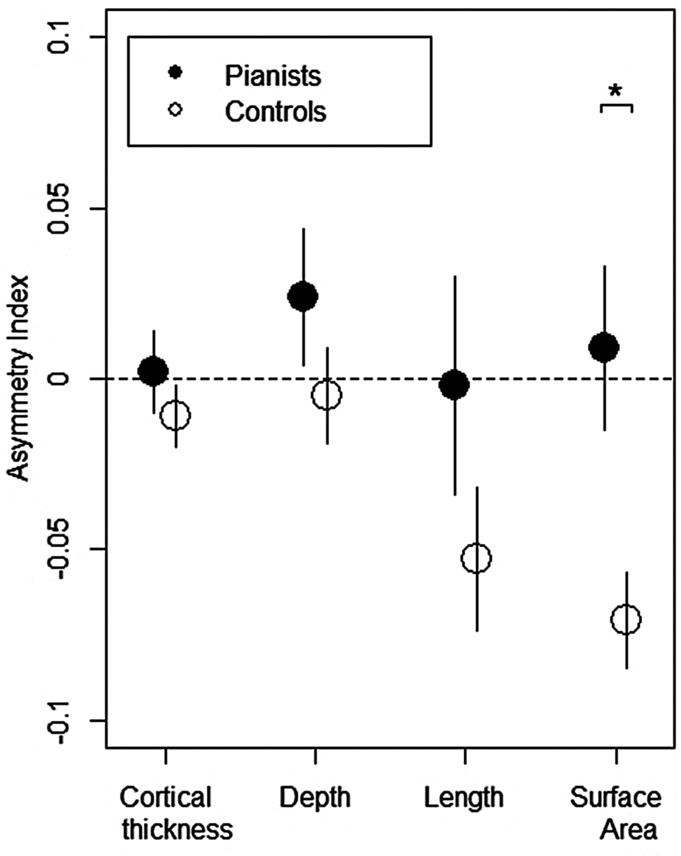
Mean AI values for the cortical thickness, depth, length, and SA of the CS in pianists (full circles) and controls (open circles). Following MANCOVA showing a main effect of group (*P* < 0.05), one-way ANCOVAs between groups revealed a significant difference in SA AI of the CS (*P* < 0.01 denoted by *). Error bars represent ± one standard error.

Mean values of the depth, length, and SA of the CS and of the thickness of the cortical mantle adjacent to the CS in the left and right cerebral hemisphere for the pianists and controls are presented in Fig. 2 and corresponding AIs are presented in Fig. 3.

One-way MANCOVA across the four AIs showed a significant main effect of group (*F*_(4, 22)_ = 3.055, *P* = 0.040, Wilks' Lambda Λ = 0.647, partial eta squared ($\eta^{ 2}_{ p}$) = 0.353), and one-way ANCOVAs showed that this group effect was statistically significant for the SA measure (*F*_(1, 25)_ = 8.105, *P* = 0.009, $\eta^{ 2}_{ p}$ = 0.245, Fig. 3), but not for depth (*F*_(1, 25)_ = 1.181, *P* = 0.288), length (*F*_(1, 25)_ = 1.735, *P* = 0.200), or thickness of the cortical mantle adjacent to the CS (*F*_(1, 25)_ = 0.855, *P* = 0.364). Application of independent samples t-tests to compare mean SA AI values between pianists and controls revealed a statistically significant difference between groups (*t*_(27)_ = 2.760, *P* = 0.010, Cohen’s *d* = 1.026), where CS SA was more symmetrical in the pianists (mean AI = 0.009, SE = 0.02, *n* = 15) compared to the controls, who showed a leftward asymmetry (mean AI = -0.071, SE = 0.01, *n* = 14).

Next a 2 × 2 mixed design MANCOVA was performed on the within-hemisphere values (i.e. of the measured values rather than the AIs). This revealed a statistically significant interaction between group and hemisphere (*F*_(4, 22)_ = 3.374, *P* = 0.027, Wilks' Lambda Λ = 0.620, $\eta^{ 2}_{ p}$ = 0.380), and subsequent 2 × 2 ANCOVAs revealed that the interaction was significant between group and hemisphere for SA (*F*_(1, 25)_ = 8.486, *P* = 0.007, $\eta^{ 2}_{ p}$ = 0.253), but not for any other measure. Paired t-tests to compare left and right hemisphere values in each group showed that whereas SA of the CS was significantly greater in the left than the right cerebral hemisphere in controls (*t*_(13)_ = -4.637, *P* < 0.001, Cohen’s *d* = 1.239), there was no significant CS SA asymmetry in pianists (*t*_(14)_ = 0.487, *P* = 0.634, Cohen’s *d* = 0.126) (see Fig. 2). Additional paired t-tests comparing within-group left and right hemisphere values showed no significant differences for depth (*t*_(13)_ = -0.355, *P* = 0.728), length (*t*_(13)_ = -2.389, *P* = 0.033, which does not reach the alpha correction for multiple comparisons), or thickness of the cortical mantle adjacent to the CS (*t*_(13)_ = -1.198, *P* = 0.252) in controls, and no significant differences for depth (*t*_(14)_ = 1.218, *P* = 0.243), length (*t*_(14)_ = -0.035, *P* = 0.973), or the thickness of the cortical mantle adjacent to the CS (*t*_(14)_ = 0.199, *P* = 0.845) in pianists.

Finally, application of independent samples t-tests to compare mean values of SA between pianists and controls in turn for each cerebral hemisphere showed a statistically significant difference in the right (*t*_(27)_ = 2.60, *P* = 0.015, Cohen’s *d* = 0.966) but not in the left cerebral hemisphere (*t*_(27)_ = 0.537, *P* = 0.596, Cohen’s *d* = 0.199) (Fig. 2). In particular, the SA of the right CS in pianists was significantly greater than in controls with no significant difference in the left CS. There were no significant differences in corresponding values in the left or right cerebral hemisphere for depth, length, or thickness of the cortical mantle adjacent to the CS (*P* > 0.05).

## Discussion

Application of an automated pipeline (BrainVISA software) for extracting and measuring the morphology of cerebral sulci on 3D MR images revealed a significant difference between pianists and controls in CS SA. In contrast to leftward CS SA asymmetry in right-handed controls, right-handed pianists had on average a much more symmetrical CS. The difference was statistically significant (Fig. 3) and was driven by a significantly larger SA of the CS in the right cerebral hemisphere in pianists compared to controls. This finding is consistent with and complements the findings of Amunts *et al*. (1997) and is interpreted to likely reflect use-dependent brain plasticity where the long-term expert, bilateral, motor practice, and performance associated with piano training has increased the representation of the non-dominant hand (and therefore SA) of right CS.

The use of objective 3D MR image analysis techniques, age-, sex-, and handedness matching of a group of pianists and controls, and use of MANCOVAs controlling for age and sex are major strengths of the present study. However, we acknowledge that the relatively small sample size of only 15 pianists and 14 controls is a limitation. This limited detailed study of potential effects such as the age of onset of musical training and age and sex of the participants. Sex differences have previously been reported in measurements of the CS, and also in the cortical thickness of a number of brain regions (Amunts *et al*., 2000; Plessen *et al*., 2014), highlighting the need to continue to control for sex in future studies of the effects of musical training on CS morphology. An additional consideration is that all of the participants recruited to the present study were right-handed, and it would be interesting to perform a similar study in left-handed pianists and controls.

The findings of the present study are interpreted as reflecting experience-dependent neuroplastic changes. However, pre-existing differences in the structure of the brain may have influenced the participants in becoming pianists. However, this interpretation is considered less likely in view of substantial evidence emerging to suggest that long-term training of particular cognitive tasks and motor behaviours leads to associated changes in brain structure (Zatorre *et al*., 2012). The question has been previously investigated for musicians cross-sectionally for both grey matter (Bailey *et al*., 2014) and white matter (Moore *et al*., 2014), including specifically with adult keyboard players (Gartner *et al*., 2013) and longitudinally with children learning to play the piano (Hyde *et al*., 2009). Furthermore, recent work using MRI diffusion tensor imaging (DTI) revealed patterns of microstructural neuroplastic change in white matter suggestive of activity-dependent myelination in response to performance of a musically-cued motor task (Moore *et al*., 2017) and has provided evidence that local changes in brain morphology may occur over relatively short time scales.

The finding of no significant asymmetry of the depth or length of CS in controls is consistent with a study of post-mortem brains by White *et al*. (1997) but is contrary to one post-mortem (White *et al*., 1994) and several MRI studies in which leftward asymmetry was reported (Amunts *et al*., 1996; Zilles *et al*., 1997; Amunts *et al*., 2000; Cykowski *et al*., 2008) and one which reported rightward asymmetry (i.e. favouring the nondominant hand) (Davatzikos and Bryan, 2002). Potential explanations include differences in the methodologies employed, effects being specific to a particular region of the CS, number of participants, or level of statistical significance reported. Regarding methodology, White *et al*. (1994, 1997) studied a block of cut tissue representing an upper region of the CS, Amunts *et al*. (1996, 1997 and 2000)restricted analysis to transverse MR images with Talairach coordinates z = 69 to 35, and Cykowski *et al*. (2008) reported significant findings in different regions of the CS for males and females. Further research should also be carried out to determine the relationship between the findings observed in the present study and the morphology of the hand knob (see Caulo *et al*., 2007), which Bangert and Schlaug (2006) reported to be more prominent in the left cerebral hemisphere of keyboard players. In future studies it will also be interesting to use BrainVISA in combination with other software such as Freesurfer (https://surfer.nmr.mgh.harvard.edu/) (Dale *et al*., 1999) as has been described by Madan (2019) and which will allow detailed study of the relationship between the morphology of the CS and volume of adjacent grey matter.

In conclusion, left-greater-than-right asymmetry of the SA of the CS, observed in controls, was not found in right-handed pianists. This group difference was due to a significantly greater SA of right CS in pianists compared to controls and is interpreted to have arisen from right-handed pianists spending years of practice developing fine motor skills using their non-dominant, left hand. The present study thus provides important corroboration of previous reports of experience dependent structural plasticity in skilled musicians and has the potential to inform future research and clinical practice in the field of motor rehabilitation.

## References

[bib3] Amunts K, Jäncke L, Mohlberg H et al. (2000) Interhemispheric asymmetry of the human motor cortex related to handedness and gender. Neuropsychologia. 38:304–12. 10678696 10.1016/s0028-3932(99)00075-5

[bib2] Amunts K, Schlaug G, Jäncke L et al. (1997) Motor cortex and hand motor skills: structural compliance in the human brain. Hum Brain Mapp. 5:206–15. 20408216 10.1002/(SICI)1097-0193(1997)5:3<206::AID-HBM5>3.0.CO;2-7

[bib1] Amunts K, Schlaug G, Schleicher A et al. (1996) Asymmetry in the human motor cortex and handedness. Neuroimage. 4:216–22. 9345512 10.1006/nimg.1996.0073

[bib4] Annett MA. (1970) Classification of hand preference by association analysis. Br J Psychol. 61:303–21. 5457503 10.1111/j.2044-8295.1970.tb01248.x

[bib5] Bailey JA, Zatorre RJ, Penhune VB. (2014) Early musical training is linked to gray matter structure in the ventral premotor cortex and auditory-motor rhythm synchronization performance. J Cogn Neurosci. 26:755–67. 24236696 10.1162/jocn_a_00527

[bib6] Bangert M, Schlaug G. (2006) Specialization of the specialized in features of external human brain morphology. Eur J Neurosci. 24:1832–4. 17004946 10.1111/j.1460-9568.2006.05031.x

[bib4_648_232821] Caulo M, Briganti C, Mattei PA et al. (2007) New morphologic variants of the hand motor cortex as seen with MR imaging in a large study population. AJNR Am J Neuroradiol. 28:1480–5. 17846195 10.3174/ajnr.A0597 PMC8134386

[bib7] Cykowski MD, Coulon O, Kochunov PV et al. (2008) The central sulcus: an observer-independent characterization of sulcal landmarks and depth asymmetry. Cereb Cortex. 18:1999–2009. 18071195 10.1093/cercor/bhm224 PMC2733306

[bib13_683_095121] Dale AM, Fischl B, Sereno MI (1999) Cortical surface-based analysis. I. Segmentation and surface reconstruction. Neuroimage. 9:179–94. 9931268 10.1006/nimg.1998.0395

[bib8] Davatzikos C, Bryan RN. (2002) Morphometric analysis of cortical sulci using parametric ribbons: a study of the central sulcus. J Comput Assist Tomogr. 26:298–307. 11884791 10.1097/00004728-200203000-00024

[bib9_468_283821] Gärtner H, Minnerop M, Pieperhoff P et al. (2013) Brain morphometry shows effects of long-term musical practice in middle-aged keyboard players. Front Psychol. 4:636. 24069009 10.3389/fpsyg.2013.00636 PMC3779931

[bib10_527_284121] Hyde KL, Lerch J, Norton A et al. (2009) Musical training shapes structural brain development. J Neurosci. 29:3019–25. 19279238 10.1523/JNEUROSCI.5118-08.2009 PMC2996392

[bib5_870_233021] Kochunov P, Rogers W, Mangin JF et al. (2012) A library of cortical morphology analysis tools to study development, aging and genetics of cerebral cortex. Neuroinformatics. 10:81–96. 21698393 10.1007/s12021-011-9127-9 PMC3471145

[bib11] Li S, Han Y, Wang D et al. (2010) Mapping surface variability of the central sulcus in musicians. Cereb Cortex. 20:25–33. 19433652 10.1093/cercor/bhp074

[bib15_636_1621536865834] Madan CR. (2019) Robust estimation of sulcal morphology. Brain Inform. 6:5. 31187294 10.1186/s40708-019-0098-1 PMC6560124

[bib14] Mangin J-F, Jouvent E, Cachia A. (2010) In-vivo measurement of cortical morphology: means and meanings. Curr Opin Neurol. 23:359–67. 20489617 10.1097/WCO.0b013e32833a0afc

[bib13] Mangin J-F, Rivière D, Cachia A et al. (2004) A framework to study the cortical folding patterns. Neuroimage. 23:S129–38. 15501082 10.1016/j.neuroimage.2004.07.019

[bib15] Moore E, Schaefer RS, Bastin ME et al. (2014) Can musical training influence brain connectivity? Evidence from diffusion tensor MRI. Brain Sciences. 4:405–27. 24961769 10.3390/brainsci4020405 PMC4101485

[bib16] Moore E, Schaefer RS, Bastin ME et al. (2017) Diffusion tensor MRI tractography reveals increased fractional anisotropy (FA) in arcuate fasciculus following music-cued motor training. Brain Cogn. 116:40–6. 28618361 10.1016/j.bandc.2017.05.001 PMC5479403

[bib17] Oldfield RC. (1971) The assessment and analysis of handedness: the Edinburgh inventory. Neuropsychologia. 9:97–113. 5146491 10.1016/0028-3932(71)90067-4

[bib18] Perrot M, Denis Rivière D, Mangin J-F (2011) Cortical sulci recognition and spatial normalization. Med Image Anal. 15:529–50. 21441062 10.1016/j.media.2011.02.008

[bib19] Plessen KJ, Hugdahl K, Bansal R *et al*.(2014) Sex, age, and cognitive correlates of asymmetries in thickness of the cortical mantle across the life span. J Neurosci. 34:6294–302. 24790200 10.1523/JNEUROSCI.3692-13.2014 PMC4004815

[bib21] Steingrüber HJ. (1971) Zur Messung der Händigkeit. Z. Zeitschrift fur Experimentelle und Angewandte Psychologie. 18:337–57. 5561973

[bib11_721_284621] Steinmetz H, Volkmann J, Jäncke L et al. (1991) Anatomical left-right asymmetry of language-related temporal cortex is different in left- and right-handers. Ann Neurol. 29:315–9. 2042947 10.1002/ana.410290314

[bib23] Sun SY, Klöppel S, Rivière D et al. (2012) The effect of handedness on the shape of the central sulcus. Neuroimage. 60:332–9. 22227053 10.1016/j.neuroimage.2011.12.050

[bib24] Toga AW, Thompson PM. (2003) Mapping brain asymmetry, Nat Rev Neurosci. 4:37–48. 12511860 10.1038/nrn1009

[bib25] White LE, Lucas G, Richards A et al. (1994) Cerebral asymmetry and handedness. Nature. 368:197–8. 8145817 10.1038/368197a0

[bib8_398_233921] White LE, Andrews TJ, Hulette C et al. (1997) Structure of the human sensorimotor system. II: Lateral symmetry. Cereb Cortex. 7:31–47. 9023430 10.1093/cercor/7.1.31

[bib12_920_285121] Zatorre RJ, Delhommeau K, Zarate JM. (2012) Modulation of auditory cortex response to pitch variation following training with microtonal melodies. Front Psychol. 3:544. 23227019 10.3389/fpsyg.2012.00544 PMC3514543

[bib27] Zilles K, Schleicher A, Langemann C et al. (1997) Quantitative analysis of sulci in the human cerebral cortex: development, regional heterogeneity, gender difference, asymmetry, intersubject variability and cortical architecture. Hum Brain Mapp. 5:218–21. 20408218 10.1002/(SICI)1097-0193(1997)5:4<218::AID-HBM2>3.0.CO;2-6

